# Clinical Analysis of stereotactic body radiation therapy using extracranial gamma knife for patients with mainly bulky inoperable early stage non-small cell lung carcinoma

**DOI:** 10.1186/1748-717X-6-84

**Published:** 2011-07-20

**Authors:** Dajun Wu, Hong Zhu, Hanjun Tang, Changlin Li, Feng Xu

**Affiliations:** 1 Department of Radiation Oncology, Cancer Center, West China Hospital, Sichuan University, Chengdu 610041, China; 2 Department of Radiation Oncology, 363 hospital, Chengdu 610041, China

## Abstract

**Purpose:**

To evaluate the clinical efficacy and toxicity of stereotactic body radiation therapy (SBRT) using extracranial gamma knife in patients with mainly bulky inoperable early stage non-small cell lung carcinoma (NSCLC).

**Materials and methods:**

A total of 43 medically inoperable patients with mainly bulky Stage I/II NSCLC received SBRT using gamma knife were reviewed. The fraction dose and the total dose were determined by the radiation oncologist according to patients' general status, tumor location, tumor size and the relationship between tumor and nearby organ at risk (OAR). The total dose of 34~47.5 Gy was prescribed in 4~12 fractions, 3.5~10 Gy per fraction, one fraction per day or every other day. The therapeutic efficacy and toxicity were evaluated.

**Results:**

The median follow-up was 22 months (range, 3-102 months). The local tumor response rate was 95.35%, with CR 18.60% (8/43) and PR 76.74% (33/43), respectively. The local control rates at 1, 2, 3, 5 years were 77.54%, 53.02%, 39.77%, and 15.46%, respectively, while the 1- and 2-year local control rates were 75% and 60% for tumor ≤3 cm; 84% and 71% for tumor sized 3~5 cm; 55% and 14.6% for tumor sized 5~7 cm; and 45%, 21% in those with tumor size of >7 cm. The overall survival rate at 1, 2, 3, 5 years were 92.04%, 78.04%, 62.76%, 42.61%, respectively. The toxicity of stereotactic radiation therapy was grade 1-2. Clinical stages were significantly important factor in local control of lung tumors (P = 0.000). Both clinical stages (P = 0.015) and chemotherapy (P = 0.042) were significantly important factors in overall survival of lung tumors.

**Conclusion:**

SBRT is an effective and safe therapy for medically inoperable patients with early stage NSCLC. Clinical stage was the significant prognostic factors for both local tumor control and overall survival. The toxicity is mild. The overall local control for bulky tumors is poor. Tumor size is a poor prognostic factor, and the patients for adjuvant chemotherapy need to be carefully selected.

## Background

About 20% to 30% of patients with non-small cell lung cancer (NSCLC) are diagnosed with early stage NSCLC [1,2]. Surgery is the standard treatment of NSCLC patients, but radiation therapy is the only chance to cure T1-T2 tumors if patient is not eligible for surgery or refuses it [3-7]. Radiotherapy (RT) can offer an alternative therapy in these cases, but the outcome with conventional RT is unsatisfactory [1,5,8-10]. However, in recent years, there are enthusiasms for stereotactic body radiation therapy (SBRT) centering on the observation that small- to medium-sized tumors can be eradicated with a noninvasive therapy because of the considerable effect, and several prospective clinical results from trials using SBRT have been published [2,7,11-15]. Since large tumor size was reported to be a predictor of poor outcome of lung cancer by many studies [7,16,17], we try to evaluate the efficacy and toxicity of 43 patients with mainly bulky early stage NSCLC who had accepted the SBRT in our institution.

## Methods

### Patient population and characteristics

Forty-three patients with mainly bulky early stage NSCLC pathologically confirmed by percutaneous lung biopsy, phlegmy cytology or fiberoptic bronchoscopy were treated using SBRT with the body gamma knife system from June 2000 to October 2008. The patient characteristics are summarized in Table 1. The Clinical staging system of lung cancer (UICC 2009 version) was adopted for this study [18]. In these 43 patients, 33 patients were considered not to be candidates for surgical resection after evaluation by thoracic surgeon because of comorbidities such as cardiovascular disease, chronic obstructive pulmonary disease and diabetes. The others refused surgical resection.

**Table 1:** Patient characteristics

Characteristic		**Patients, No**.
Gender	Male	32
	Female	11
Age	Median(range),y	69(46~81)
	≤70	23
	>70	20
Karnofsky performance status	70	7
	80	19
	90	17
Comorbidities	Yes	33
	No	10
Type	Central	8
	Periphery	35
Histology	Squamous cell carcinoma	17
	Adenocarcinoma	14
	Adenosquamouscarcinoma	1
	unspecified	11
Clinical stage	i	23
	ii	20
Tumor size	≤3 cm	4
	3-5 cm	20
	5-7 cm	10
	>7 cm	9
Dose/fraction (50% isodose line)	<6Gy	27
	≥6Gy	16

### Radiotherapy equipment

Patients were treated using the stereotactic gamma-ray whole-body therapeutic system (body gamma-knife) developed by OUR International Technology & Science Co., Ltd. (Shenzhen, China). The body gamma knife uses rotary conical surface focusing to focalize 30 Co-60 sources with total activity of 8500 Ci, the focal dose rate at the initial source setting was 3 Gy/min. The body gamma knife consists of a radiation source, collimator, and treatment bed. The head of radiation source is an iron ball rind with 30 Co-60 sources scattered throughout the cavity of the primary collimator. The source body rotates horizontally around the central axis with the 30 bundles of gamma ray directed toward a focal target. In the present study, three groups of chamber with collimator aperture diameters of 3 mm, 12 mm, and 18 mm, respectively, were used; the full width at half-height of the dose-field range at the target was 10 mm, 30 mm, and 50 mm, respectively. As the aperture diameter of the collimator decreased, the density of the distributed dose increased, and the periphery dose decreased. Three groups of terminal collimators with different apertures direct the focusing of the radials. Target volume of 1-10 cm in diameter could be treated using a combination of collimators with different aperture diameters. Finally, the treatment bed can move in X, Y, and Z directions and can automatically adjust the target to the focal point of the radials.

### Treatment planning and delivery

Supine or prone position was selected according to diagnostic chest CT scan, and each patient could keep the posture for 30 minutes. All patients were immobilized using a stereotactic body frame with a vacuum pillow to create reproducible immobilization. Abdominal clamping pressure was applied using a diaphragm control device. The planning CT was scanned with 3 mm slice throughout the tumor, and 5 mm slice in other areas of the thorax and upper abdomen. After the scan was finished, the positional parameters were recorded in order to repeat the position when the patient was irradiated. The images of CT simulation were then imported into the treatment planning system (OUR WB-GR TPS99). Reconstructions were performed on a three-dimensional conformal radiotherapy planning algorithm. The Dose-Volume Histogram (DVH) was used to calculate V20 (percent volume of total lung receiving 20 Gy) and the doses of other OARs. The gross tumor volume (GTV) was the primary tumor. The clinical target volume (CTV) was identical to GTV. The planning target volume (PTV) was created using pulmonary window which allowed a 1.0 cm margin around the CTV. A radiation dose was prescribed to the 50% isodose line. Three-dimensional imaging of isodose coverage of GTV and PTV was used to select aperture diameter, the number and location of target iso-center depended on the size and shape of the target volume. The total dose was between 34 and 47.5 Gy (at 50% isodose line) which represents between 56 and 80 Gy calculated in BED_10_(biological equivalent) dose using the LQ (Linear quadratic) model and α/β equal to 10 for the tumor. The median value of BED at the isocenter (at 100% isodose line) was 193.2 Gy (range, 184.8~201.6 Gy) in 4 patients with tumor size of ≤3 cm, 194.8 Gy (range, 142.8~240.0 Gy) in 24 patients with tumor size of 3~5 cm, 165.5 Gy (range, 142.8~212.2 Gy) in 10 patients with tumor size of 5~7 cm and 144.0 Gy (range, 142.8~184.8 Gy) in 9 patients with tumor size of >7 cm.

The strategy was to achieve a dose volume constraint for lung of V20 Gy <35%, a maximal dose < 50 Gy of the BED to the esophagus and < 45 Gy to the spinal cord. Radiotherapy was delivered over 1~2 weeks. 4 patients had received prophylaxis irradiation of the mediastinum with doses of 40~46 Gy in 20~23 fractions, of which 3 patients had stage T2b, 1 patient had stage T3.

### Chemotherapy

13 patients had received 2~4 cycles chemotherapy with NVB (25 mg/m^2^, d1,d8) and DDP (75 mg/m^2^, d1, repeated every 3 weeks). In these 13 patients, 1 patient was staged T1b (≤ 3 cm), 5 patients staged T2a (3~5 cm), 4 patients staged T2b (5~7 cm) and 3 patients staged T3 (> 7 cm). The mean age of the patients was 69 years (range 46~76). 6 of the patients had co-morbidities.

### Evaluation of therapeutic efficacy and toxicity

The short-term therapeutic effects of local tumor control was classified as complete response (CR), partial response (PR), stable disease(SD), or progressive disease(PD) as judged according to CT image. According to Response Evaluation Criteria in Solid Tumors (RECIST) (WHO 2000 version) [19], a CR was defined as complete disappearance of all measurable disease for 4 weeks, a PR was defined as a 50% reduction in the sum of the perpendicular diameters of all measurable lesions for 4 weeks, and PD was defined as a 25% increase in the sum of the perpendicular diameters of all measurable lesions and new lesions that developed for 4 weeks. Patients whose disease did not meet the criteria for either a PR or progressive disease were classified as having stable disease for 4 weeks. Overall efficacy consisted of CR and PR evaluated at four weeks after treatment was finished.

The major indexes of long-term effects were survival and local control. Local recurrence was judged according to chest CT image, PET-CT image, or biopsy. The time of local control was defined as the duration from the beginning date of SBRT to the date of local recurrence. The time of survival was duration from the beginning date of SBRT to the date of follow-up for surviving patients or to the date of death.

The radiation reaction was classified as early or late adverse effects in lung, skin, esophagus and bone marrow according to NCI-CTC 3.0 version [20]. The early adverse effects were defined to occur within the first 90 days after the beginning date of SBRT, and the late adverse effects occur beyond the first 90 days after the beginning date of SBRT.

### Follow-up

The follow-up duration was defined as the time from the beginning date of SBRT to the last date of follow-up for surviving patients or to the date of death. The last date of follow-up was in June 30th, 2009. The median duration of follow-up was 22 months (range, 3~102 months).

### Statistical analysis

The short-term therapeutic effects of local tumor control were evaluated using direct method. The SPSS software program (version 16.0; SPSS Inc, Chicago, IL) was used for all statistical analyses. The Kaplan-Meier method was used to evaluate the survival and local control rates. The survival and local control durations were evaluated from the day of treatment. The log-rank test was used to compare the different levels of a factor. Cox Regression model was used for multivariate analysis of local control and survival. *P *< 0.05 was considered statistically significant.

## Results

### Response rate

The short-term effects of local control were evaluated at four weeks after treatment finished. The CR rate in the primary tumor was 18.60% (8/43), Also, The PR rate in the primary tumor was 76.74% (33/43), 2 patients had stable disease. None of the patients had progressive disease. The overall response rate (CR + PR) in the whole study group was 95.35%.

### Pattern of failure

**Figure 1:**
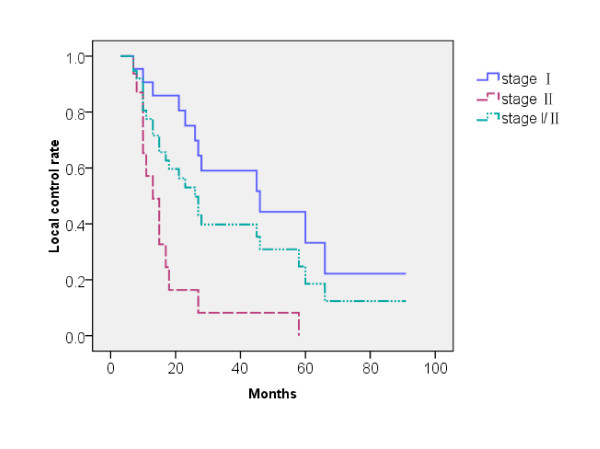
**The local control of patients with different stages**.

**Figure 2:**
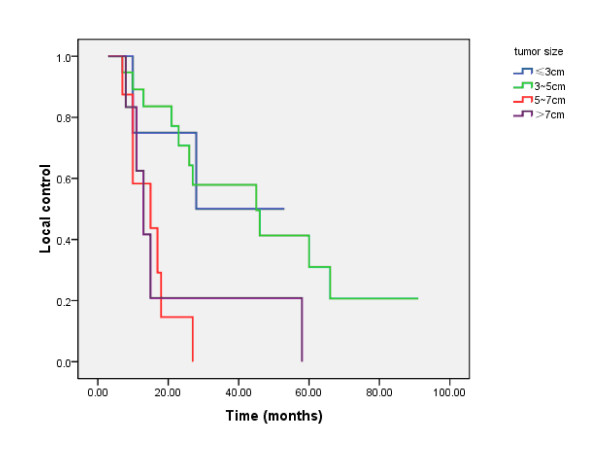
**The local control of patients with different tumor sizes**.

The 1-, 2-, 3-, and 5-year local control rates (defined as no progressive disease at the primary treatment site) in all the patients were 77.54%, 53.02%, 39.77%, 15.46% (Figure 1), respectively. The 1-, 2-, 3-, and 5-year rates of local control in those with Stage I disease were 91%, 75%, 58%, 44%, respectively, and 49%, 16%, 8%, 8%, respectively, in those with Stage II disease (Figure 1, *p *= 0.000). The 1- and 2-year local control rates were 75% and 60% for tumor ≤3 cm; 84% and 71% for tumor sized 3~5 cm; 55% and 14.6% for tumor sized 5~7 cm; and 45%, 21% in those with tumor size of >7 cm (Figure 2). 25 patients had a local recurrence (Table 2). The median duration of recurrence was 26 months (range, 3~91 months), 5 of 25 patients had a local recurrence over 36 months of treatment. The rates of metastasis were 25%, 65%, 40%, 89% in patients with tumor size of ≤3 cm, 3-5 cm, 5-7 cm, >7 cm, respectively.

**Table 2:** the patterns of failure for tumor of different sizes

Tumor size	≤3 cm	3~5 cm	5~7 cm	>7 cm
Patients No.	4	20	10	9
Local recurrence	2	11	7	5
metastasis	1	13	4	8

### Overall survival

**Figure 3:**
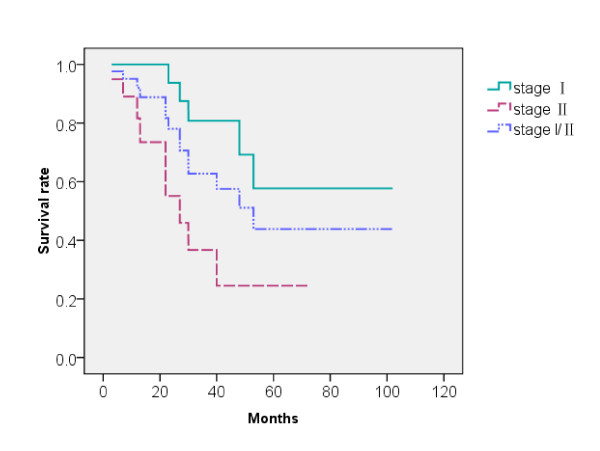
**The survival of patients with different stages**.

The median survival time was 53 months (range, 3~102 months). The 1-, 2-, 3-, and 5-year rates of overall survival in the whole group were 92.04%, 78.04%, 62.76%%, 42.61% (Figure 3), respectively. The 1-, 2-, 3-, and 5-year overall survival rates in patients with Stage I disease were 100%, 94%, 81%, 58%, respectively, and 82%, 55%, 37%, 24%, respectively, in patients with Stage II disease (Figure 3, *p *= 0.005). Overall, 14 patients died: 3 died of local recurrence, and 8 died of regional lymph node or distant metastases. 2 died of systemic failure. 1 patient with chronic obstructive pulmonary disease (COPD) died of severe lung infection.

Patients with local recurrence, regional lymph node or distant metastases had accepted the second SBRT because the toxicity of the first SBRT was mild. 22 of 25 patients with local recurrence had the second SBRT. After the treatment, 2 patients achieved CR; 15 patients had PR; 4 patients had SD; and 1 patient had PD. The overall response rate was 77.3%. Three patients didn't receive second SBRT, one of them received 2 cycles of systemic combined chemotherapy with NVB and DDP. One patient with one paratrachea lymph node metastasis received SBRT. Five patients received SBRT of lung metastases. One patient received SBRT for bilateral adrenal metastases. The rest of patients with tumor progression were only given symptom relieving treatment and supportive treatment.

### Factors influencing outcome of treatment

The Univariate analysis showed that clinical stage and tumor size had significant impact on local tumor control (Table 3, 4) (p < 0.05); but multivariate analysis showed only clinical stage had significant impact on local tumor control (Table 5) (p < 0.05).

**Table 3:** Univariate analysis of local tumor control and overall survival (log rank of Kaplan-Meier method)

Characteristic		**Patients, No**.	Local control		Overall survival	
			
			*Χ^2^*	*P*	*Χ^2^*	*P*
Gender	Male	32	1.054	0.305	2.671	0.102
	Female	11				
Age	≤70	23	0.832	0.362	0.116	0.733
	>70	20				
Karnofsky performance status	70	7	1.584	0.453	1.769	0.413
	80	19				
	90	17				
Comorbidities	Yes	33	0.442	0.506	0.007	0.932
	No	10				
Type	Central	8	0.427	0.514	0.173	0.678
	Periphery	35				
Histology	Squamous cell carcinoma	17	0.696	0.874	0.975	0.807
	Adenocarcinoma	14				
	Adenosquamouscarcinoma	1				
	Unspecified	11				
Clinical stage	I	23	14.888	0.000	7.926	0.005
	II	20				
Tumor size	≤3 cm	4	12.824	0.005*	5.361	0.147
	3-5 cm	20				
	5-7 cm	10				
	>7 cm	9				
Dose/fraction (50% isodose line)	<6Gy	27	1.337	0.248	0.979	0.322
	≥6Gy	16				
BED (50% isodose line)	<60Gy	17	0.550	0.458	0.033	0.856
	≥60Gy	26				
Chemotherapy	no	30	2.194	0.139	6.000	0.014
	yes	13				

Abbreviation: BED: biological effective dose.

*: pairwise comparisons of local control of different size tumors.

**Table 4:** Pairwise comparisons of local control of different size tumors

	Tumor size	≤3 cm		3~5 cm		5~7 cm		>7 cm	
		
Method		Chi-Square	Sig	Chi-Square	Sig	Chi-Square	Sig	Chi-Square	Sig
Log Rank (Mantel-Cox)	≤3 cm			.002	.961	4.638	.031	.895	.344
	
	3~5 cm	.002	.961			10.977	.001	4.541	.033
	
	5~7 cm	4.638	.031	10.977	.001			.108	.742
	
	>7 cm	.895	.344	4.541	.033	.108	.742		

**Table 5:** Multivariate analysis of local tumor control (Cox Regression method)

Factor	B	SE	Wald	df	**Sig**.	Exp(B)	95.0% CI for Exp(B)	
							Lower	Upper
Clinical stage	1.557	.444	12.323	1	.000	4.745	1.989	11.320

Univariate analysis (Table 3) and multivariate analysis (Table 6) showed that both clinical stage and chemotherapy were significant prognostic factors of overall survival (p < 0.05).

**Table 6:** Multivariate analysis of overall survival (Cox Regression method)

factor	B	SE	Wald	df	**Sig**.	Exp(B)	95.0% CI for Exp(B)	
							Lower	Upper
Clinical stage	1.402	0.575	5.939	1	.015	4.064	1.316	12.553
Chemotherapy	1.297	.637	4.141	1	.042	3.659	1.049	12.760

### Toxicity

The radiation-induced side effects of 43 patients after SBRT were mild (Table 7), mostly grade 1~2, no grade 3 or above. None of the patients discontinued treatment because of radiation toxicities.

**Table 7:** Radiation-induced side effects based on NCI-CTC 3.0 version.

Side effect	**Early No**.			**Late No**.		
	
	Grade			Grade		
	
	1	2	3	1	2	3
Pneumonitis	6	4	0	2	0	0
Esophagitis	3	4	0	0	0	0
Hematologic	8	2	0	0	0	0
Dermatitis	6	2	0	1	0	0

## Discussion

SBRT can improve cure rate of tumor and reduce radiation-induced side effects by overcoming insufficiency of conventional fractionated radiotherapy [21-24]. SBRT can make sharp gradient of dose fall- off to improve normal structure sparing and achieve highly conformal dose distribution in tumor target by using 3D-planning technique. Based on this technique, SBRT allows for ab**l**ative doses to be delivered over a few fractions within 2 weeks. In recent years, SBRT has been widely used in clinical practice and become a major treatment modality for early stage NSCLC to obtain excellent outcome [14,15,25-29].

In our study, most of patients had bulky lesions. The local control was poor, especially for these with lesions over 5 cm. Based on analysis of prognostic factors of local tumor control in this study, both clinical stage and tumor size were significant factors in Univariate analysis (Table 3, 4) (p < 0.05); however, clinical stage was the only significant prognostic factor in multivariate analysis (Table 5) (p < 0.05). The pair wise comparisons of local control in different tumor size demonstrated that local tumor control rate for tumor sized 3~5 cm was significantly better than that of 5~7 cm and >7 cm (Table 4) (p < 0.05). However, the local tumor control rate in Xia's study was 95% [2], better than that of our study. In Xia's study, 18 patients had tumor size ≤3 cm, 21 had tumor size of 3~5 cm, and 4 had had tumor size >5 cm and with tumor BED value of 75 Gy (at 50% isodose line). However, in our study, only 4 patients had tumor size ≤3 cm, 20 patients had tumor size of 3~5 cm, and 19 patients had tumor size >5 cm; and the tumor mean BED value was 62.86 Gy (range, 56~80 Gy, 50% isodose line). It was considered that the percent of different size tumors and tumor BED might explain the better local tumor control rate of Xia's study as compared to that of our study. The BED at the isocenter (at 100% isodose line) was high enough in this study; however, the local control was poor, especially for those with lesions over 5 cm. Fakiris et al[25] had conducted a prospective phase II trial of SBRT to treat 70 medically inoperable NSCLC patients and achieved a 3-year local control of 88.1%. However, 50% of their patients had stage T1 lesions and none of their patients had tumor size larger than 7 cm. Chi et al[26] had made a systemic review on the patterns of failure following stereotactic body radiation therapy in early stage non-small cell lung cancer; it was found that tumor centre or periphery BED was important parameter influencing local tumor control rate and larger doses should be delivered for T2 tumor. Therefore, more works needs to be done in future clinical study to explore the optimal doses should be delivered for tumors of different size.

Based on the analysis of prognostic factors of overall survival rate in this study, clinical stage and chemotherapy were significant factors in multivariate analysis (Table 6) (p < 0.05). The mortality of the patients with stage II was 4.064 times higher than that of the patients with stageI (p = 0.015). Systemic chemotherapy increased the risk of death of the patients with early stage NSCLC in our study, the mortality of the patients with chemotherapy was 3.659 times higher than that of the patients without chemotherapy (p = 0.042). In these 13 patients receiving chemotherapy, the number of cases with tumor size ≤ 3 cm, 3~5 cm, 5~7 cm, > 7 cm were 1 (7%), 5 (39%), 4 (31%), 3 (23%), respectively. Based on our study, adjuvant chemotherapy may be helpful in the setting of T3 lesions in some patients. The reason that chemotherapy decreased the overall survival of the patients in this study may be that most patients in this cohort are elderly, with the mean age of 69 years. It's hard for them to recover from the influence of chemotherapy like bone marrow suppression, gastrointestinal reaction and so on. So we think the patients for adjuvant chemotherapy need to be carefully selected. The patients with local tumor recurrence, or regional lymph node, or distant metastasis were given second SBRT because the radiation-induced side effects were mild. Siva et al[30] reported the local tumor control rate of patients with limited lung metastasis who received SBRT or SRS were 77.9% or 78.6% in 2 years after treatment, respectively; the toxicity was low, the rate of the toxicity above grade 3 was 4% or 2.6%, respectively; the overall survival rate was 53.7% or 50.3%, respectively. So the second SRT could be the important prognostic factor of overall survival rate in our study, which is worth further evaluating in future clinical study.

In this study, the toxicity (Table 7) was mild. No severe toxicity was observed during the whole treatment process. This can be due to the low peripheral dose prescribed in this study.

There were some limitations of our study. The dose fractionation schedule used was not uniform, and tumor motion is not well controlled without utilizing modern 4D CT to generate an IGTV based on MIP reconstruction. Also, the study is retrospective, and a prospective study in the future will need to be conducted.

## Conclusion

SBRT is an effective and safe therapy for patients with medically inoperable early stage NSCLC. Clinical stage was the significant prognostic factors for both local tumor control and overall survival. The toxicity of SBRT is mild. The overall local control for bulky tumors is poor. Tumor size is a poor prognostic factor, and the patients for adjuvant chemotherapy need to be carefully selected.

## Competing interests

The authors declare that they have no competing interests.

## Authors' contributions

DJW and HZ carries out the design of the study and draft the manuscript; HJT and CLL worked on analysis of data and helped collection of data. FX contribute to the conception of this study and the final approval of the version to be published. All authors read and approved the final manuscript.
